# The optimal timing of breast cancer surgery after COVID-19 infection: an observational study

**DOI:** 10.1186/s12885-024-13080-1

**Published:** 2024-11-03

**Authors:** Zhao Bi, Wei-Hao Cheng, Wen-Hao Zheng, Tong-Yue Ren, Peng Chen, Yan-Bing Liu, Peng-Fei Qiu, Wei-Li Wang, Yong-Sheng Wang

**Affiliations:** 1 Shandong Cancer Hospital and Institute, Shandong First Medical University, Shandong Academy of Medical Sciences, Ji Yan Road 440, Jinan, 250000 Shandong Province People’s Republic of China; 2 Rizhao Central Hospital, Rizhao, 250000 Shandong People’s Republic of China

**Keywords:** Breast neoplasms, COVID-19, Postoperative complications, Surgery

## Abstract

**Purpose:**

It is controversial for the optimal time of breast cancer surgery after COVID-19 infection. Purpose was to assess the risk of postoperative complication in breast cancer patients with COVID-19 infection, in order to select optimal surgery timing after COVID-19 infection.

**Methods:**

Breast cancer patients infected with COVID-19 and performed surgery between December 20th, 2022 to March 20th, 2023 were included in this prospective study (*n* = 577). Patients performed surgery between May 1, 2019 to October 1, 2019 were listed as control group (*n* = 329). They had not been infected with COVID-19 before surgery. Patients were grouped by time of surgery relative to COVID-19 infection. Database was evaluated using logistic regression.

**Results:**

Patients infected with COVID-19 had a higher incidence of complications after surgery compared to that not-COVID-19 infection (6.59% vs. 3.04%). Multivariable logistic analysis demonstrated that timing of surgery was associated with complications (OR = 4.253; 95% CI: 0.855–21.153, *P =* 0.044). Patients performed surgery within 2 weeks after COVID-19 infection had the highest rates of complication (17.65%) when compared with other groups, while the incidence was decreased into 5.51% when surgery 2 weeks or more after COVID-19 infection. With a median follow-up was 10 months, all patients with complications were recovered without serious complications or death, which had no adverse effect on subsequent anti-tumor therapy.

**Conclusions:**

It needs to be cautious when breast cancer surgery was performed within 2 weeks after COVID-19 infection. Although the incidence of complications in patients undergoing surgery 2 weeks after COVID-19 infection is still slightly high, surgery might be recommended considering urgency of treatment, good prognosis of complications and the lack of influence on subsequent adjuvant therapy.

## Introduction

As of May 2023, the WHO determined that coronavirus disease 2019 (COVID-19) is now an established and ongoing health issue which no longer constitutes a public health emergency of international concern [1]. Patients with cancer are more susceptible to COVID-19 infection because of the systemic immunosuppressive state caused by the malignancy and anticancer treatments, such as chemotherapy or surgery [2]. Breast cancer has accounted for almost one-third of all new cancer diagnoses in women [3]. Therefore, there are extremely large number of breast cancer patients infected with severe acute respiratory syndrome coronavirus 2 (SARS-CoV-2). During the COVID-19 pandemic, breast cancer surgery was often delayed. In the era of “Post COVID-19 Pandemic”, the gradual stabilization of COVID-19 pandemic shifted the focus of doctors and patients towards the preoperative evaluation of coronavirus-infected patients, the timing of surgery, and the prevention of postoperative complications [4].

The pre-operative COVID-19 infection was shown to be associated with significantly increased risks of postoperative complication, such as pneumonia, respiratory failure, arrhythmias, and deep vein thrombosis (DVT) [5–8]. The results from COVID-19 Surg collaboration showed that in patients diagnosed with SARS-CoV-2 infection before surgery who underwent surgery within 7 weeks of infection, the mortality rate was 3.6-4.1% at 30 days after surgery [9]. And the mortality rate decreased into 1.5% after 7 weeks of infection, which is same as uninfected people. A multi-center study from the United States showed that patients who underwent surgery within 4 weeks after infection had a significantly increased risk of postoperative complications [10]. The incidence of postoperative complications would decrease when patients underwent surgery at 4–8 weeks after infection. However, the different surgical types may affect the occurrence of postoperative complications, and the postoperative complications of breast cancer surgery might also differ from other types of surgery [11].

Therefore, it is controversial for the optimal time of breast cancer surgery after COVID-19 infection. And breast cancer patients infected with COVID-19 require individualized preoperative evaluation, and the influence of different factors need also to be comprehensively evaluated to select the optimal time for surgery. The objective of this study is to explore the optimal breast cancer surgery timing after COVID-19 infection.

## Methods

### Ethical statement

The study protocol was approved by the Institutional Review Board of the Shandong Cancer Hospital (No. SDTHEC20220324) and the study was performed in accordance with the principles of the Declaration of Helsinki. All methods were performed in accordance with the relevant guidelines and regulations. Written informed consent was obtained from patients for publication and any accompanying images.

### Study design

This study was a single-center, prospective observational study. According to the time of surgery after COVID-19 infection, patients were categorized into six groups. (1) The “peri-COVID-19” group: surgery performed 0 to 2 weeks after COVID-19 infection. (2) Patients underwent surgery between 3 and 4 weeks after COVID-19 infection. (3) Patients underwent surgery between 5 and 6 weeks COVID-19 infection. (4) Patients underwent surgery between 7 and 8 weeks after COVID-19 infection. (5) The “post-COVID-19” group: surgery performed > 8 weeks after COVID-19 infection. (6) The “pre-COVID-19” group was selected as our control group because they had not been infected with SARS-CoV-2 before surgery and any 30-day postoperative complication they developed could not be attributed to sequelae of COVID-19 infection.

### Patients

Before December 2022, the COVID-19 was classified as category A infectious disease. After December 2022, our country began to downgrade COVID-19 as category B infectious disease [12]. Our government no longer controlled the infected population, so the incidence of COVID-19 has soared like other area of the world after our country began to downgrade COVID-19 from category A infectious disease to category B. Therefore, breast cancer patients with a confirmed COVID-19 diagnosis between December 20th, 2022 to March 20th, 2023 were included in the study. The number of patients without COVID-19 infection in our hospital was very small during the same period, so we chose the cases from May 1, 2019 to October 1, 2019 as control group. These patients had not been infected with SARS-CoV-2 before surgery. And any postoperative complication could not be attributed to sequelae of SARS-CoV-2 infection.

Adult women were included in this study if they (1) had histologically confirmed invasive breast carcinoma; (2) underwent major elective operations in breast cancer, which include mastectomy or breast-conserving surgery. Exclusion criteria included patients who underwent neoadjuvant therapy, concurrent cancer, bilateral breast cancer, or distant metastases.

### Assessments

The date that patients infected with COVID-19 in this study cohort was defined as the date of the first COVID-19 Reverse Transcription Polymerase Chain Reaction test [13]. COVID-19 severity was categorized as mild, moderate (pneumonia) and severe (e.g., requiring supplemental oxygen) [12, 13]. All patients were vaccinated against the COVID-19 (Inactivated vaccine against COVID-19) before surgery. There were three manufacturers producing the vaccine (Beijing Bio-pharmaceutical Co., LTD; Beijing Sinovac Biological Products Co., LTD; Sinopharm Zhongsheng Beijing Company).

### Complications

Breast cancer surgery is less invasive and has less complicated. In our study, the complications after breast cancer surgery were only deep vein thrombosis (DVT) and incision complication, and there was no other postoperative complication. So, the primary endpoint was the incidence of overall complication in the 6 months after surgery. The clinical presentation of DVT varies with the extent and location of a thrombus. The cardinal signs and symptoms of DVT include asymmetrical swelling, warmth, or pain in an extremity. The ultrasound examination indicated emboli formation in the blood vessels. The incision complication includes incision bleeding and dehiscence in our study.

We analyzed the rate of postoperative complication between different group. At the same time, we made follow-up of patients with postoperative complication, and analyzed the outcome of patients.

### Statistical analysis

The association of different clinic-pathological variables (T stage, N stage), timing of surgery after COVID-19 infection, and the risk of postoperative complication was analyzed. Pearson chi-square test or Fisher exact test was used to perform univariate analysis on categorical variables. Multivariable logistic regression analysis was conducted to identify the independent predictive factors by using backward stepwise analysis.

Statistical analyses were performed using SPSS Statistics 22.0 software (IBM Corporation, Armonk, NY, USA) and R version 3.3.3 software (The R Foundation for Statistical Computing, Austria, Vienna). A *p* < 0.05 was considered statistically significant.

## Results

### The characteristics of enrolled patients

**Fig. 1:**
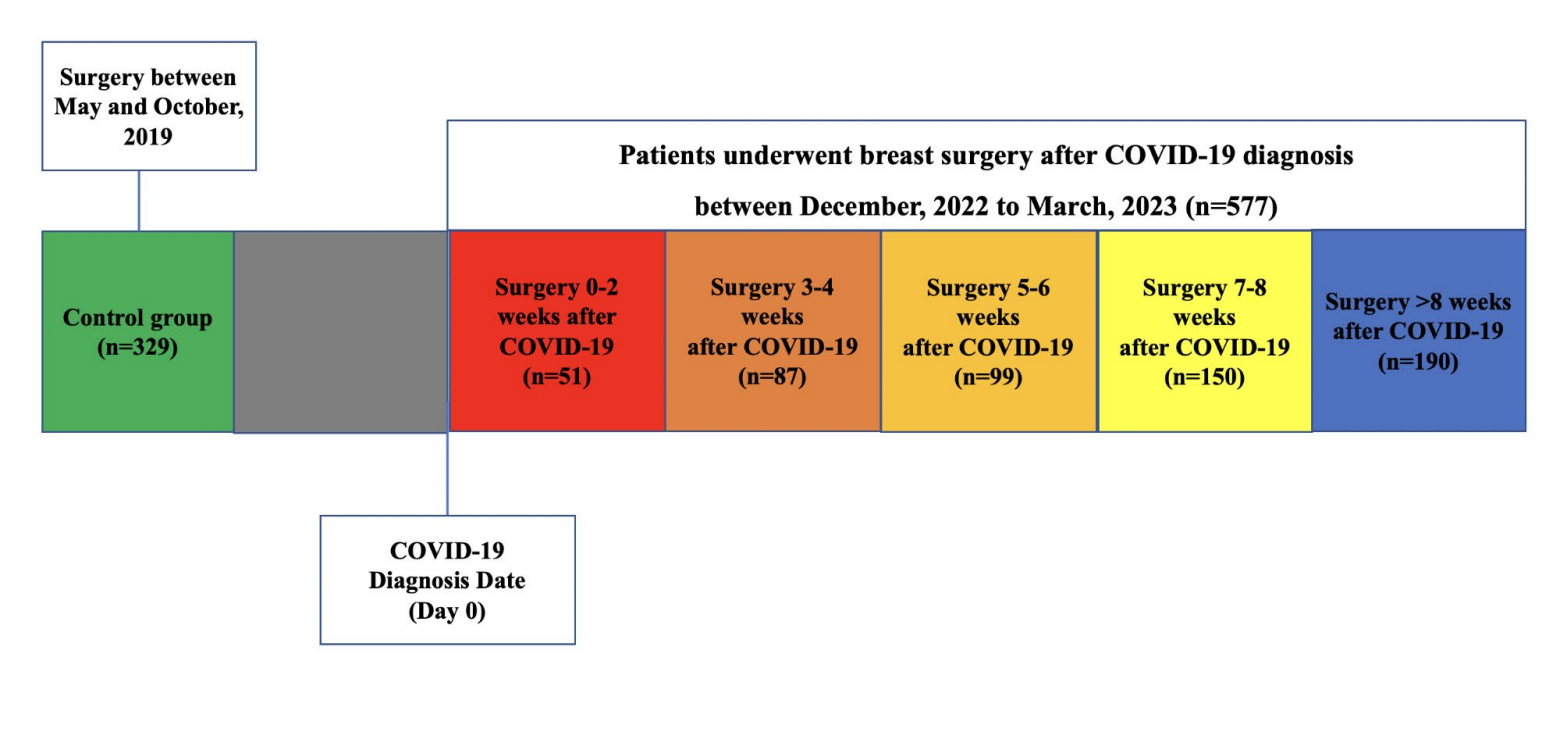
The consort diagram of the study

The consort diagram of the study was illustrated in Fig. 1. From December 20th, 2022 to March 20th, 2023, there were 640 patients underwent surgery after COVID-19 infection. After exclude patients who underwent neoadjuvant therapy (*n* = 63), 577 patients performed surgery after COVID-19 infection were included in this retrospective study. Of these patients infected with SARS-CoV-2, there were 51, 87, 99, and 150 patients underwent surgery 0–2, 3–4, 5–6, and 7–8 weeks after their COVID-19 diagnosis date, respectively. And 190 patients underwent surgery 8 weeks or more after their COVID-19 diagnosis date. There were 329 patients underwent surgery between May 1, 2019 to October 1, 2019 which were defined as control group. They had not been infected with SARS-CoV-2 before surgery. Therefore, any postoperative complication could not be attributed to sequelae of SARS-CoV-2 infection. The basic clinical and pathologic characteristics of enrolled patients were summarized in Table 1.

**Table 1:** The clinical characteristics of patients

Characteristic	Control group		Peri-Covid-19 Surgery 0-2wk After Covid-19		Early Post-Covid-19 Surgery 3-4wk After Covid-19		Early Post-Covid-19 Surgery 5-6wk After Covid-19		Early Post-Covid-19 Surgery 7-8wk After Covid-19		Late Post-Covid-19 Surgery > 8wk After Covid-19	
	No.	%	No.	%	No.	%	No.	%	No.	%	No.	%
Age (median)	49		49		49		45		44		50	
Clinical N stage												
cN_0_	226	68.7	33	64.7	39	44.8	76	76.7	90	60.0	107	56.2
cN_1_	72	21.9	15	29.4	30	34.6	7	7.1	45	30.0	50	26.6
cN_2_	13	3.9	3	5.9	9	10.3	10	10.1	12	8.0	21	10.8
cN_3_	18	5.5	0	0	9	10.3	6	6.1	3	2.0	12	6.4
Clinical T stage												
cT_1_	206	62.6	21	41.2	30	34.6	61	61.6	69	46.0	103	54.3
cT_2_	116	35.3	27	52.9	48	55.1	26	26.3	66	44.0	79	41.5
cT_3_	3	0.9	0	0	9	10.3	9	9.1	3	2.0	5	2.6
cT_4_	4	1.2	3	5.9	0	0	3	3.0	12	8.0	3	1.6
Breast surgery												
Mastectomy	259	78.7	27	52.9	66	75.8	60	60.6	111	74.0	119	62.6
BCS	70	21.3	24	47.1	21	24.2	39	39.4	39	36.0	71	37.4
Concomitant syndromes												
hypertension	33	10.0	6	11.8	15	17.2	9	9.1	18	12.0	19	10.0
diabetes	7	2.1	3	5.9	0	0	1	3.0	6	4.0	10	5.2
liver disease	20	6.1	3	5.9	0	0	0	0	0	0	2	1.1
stroke	2	0.6	0	0	0	0	0	0	0	0	2	1.1
CAD	1	0.3	0	0	3	3.4	0	0	0	0	3	1.5
COVID-19 severity												
Mild/moderate	NA	NA	48	94.1	84	96.5	99	100	147	98.0	189	99.5
severe	NA	NA	3	5.9	3	3.5	0	0	3	2.0	1	0.5

BCS: breast conserving surgery; CAD: coronary artery disease

The majority of patients had mild COVID-19 with only 8 patients (1.38%) having moderate COVID-19 (pneumonia). However, these 8 patients with mild COVID-19 did not have the risk of complication after surgery. The majority of patients had temperature below 39℃, 7.2% having temperature more than 39℃. There were 5, 10, 8, 10, 9 patients underwent surgery 0–2, 3–4, 5–6, 7–8, and > 8 weeks after their COVID-19 diagnosis date, respectively. Among 5 patients with high fever symptoms in 0–2 weeks group, the interval between high fever and operations was at least more than 1 week. In our center, for patient has a high fever during surgical instruction, considering the possible impact on anesthesia, we would advise the patient to suspend the operation until the high fever period is over. Across all groups, the most common comorbidities were obesity, diabetes and hypertension. The most common operation for both the pre-COVID-19 group and post-COVID-19 groups was mastectomy.

### The incidence of overall complication after surgery

The incidence of postoperative complication for each group are detailed in Table 2. The overall complication (6.59%) after COVID-19 infection was much higher than that in patients with pre-COVID-19 infection (3.04%, *P* = 0.033). Patients with peri-operative SARS-CoV-2 infection had the highest incidence of overall complication (17.65%) when compared with other groups, while the incidence was decreased into 5.51% when surgery 2 weeks or more after their COVID-19 diagnosis date. Patients with surgery 3–4, 5–6, and 7–8 weeks after their COVID-19 diagnosis date had 4.59%, 8.08%, and 8.00% of overall complication risk, respectively. Patients with surgery 8 weeks or more after their COVID-19 diagnosis date had a similar incidence of postoperative complication (2.63%) compared with the pre-COVID-19 group.

**Table 2:** The postoperative complications after breast cancer surgery in different groups

Groups	DVT		Incision complication		Overall complication	
	No.	%	No.	%	No.	%
Control group (*n* = 329)	4	1.21	6	1.82	10	3.04
Surgery 0–2 week After COVID-19 (*n* = 51)	6	11.76	3	5.88	9	17.65
Surgery 3–4 week After COVID-19 (*n* = 87)	3	3.45	1	1.15	4	4.59
Surgery 5–6 week After COVID-19 (*n* = 99)	3	3.03	5	5.05	8	8.08
Surgery 7–8 week After COVID-19 (*n* = 150)	6	4.00	6	4.00	12	8.00
Surgery > 8 week After COVID-19 (*n* = 190)	3	1.58	2	1.05	5	2.63

DVT: deep vein thrombosis

The results of univariate analysis showed that the time period of surgery was associated with the incidence of overall complication significantly. The incidence of overall complication in patients with peri-COVID-19 infection was significantly higher than other groups (Table 3). Multivariable logistic regression analysis demonstrated that the time period of surgery was the independent predictor of overall complication (OR = 4.253; 95% CI: 0.855–21.153, *P =* 0.044) (Table 4). After adjustment for patient characteristics and type of surgery, peri-COVID-19 patients had the highest risk of developing postoperative complication (OR = 6.836; 95% CI: 1.691–27.632, *P =* 0.007) when compared to other groups. Compared with pre-COVID-19 patients, patients infected SARS-CoV-2 within 8 weeks also had a higher incidence of complication after surgery (OR = 1.786; 95% CI: 1.163–2.808, *P* = 0.077), but there was no significant difference. Notably, patients with surgery 3–8 weeks after their COVID-19 diagnosis date also did have a higher risk of postoperative complication when compared to pre-COVID-19 patients. However, there was no difference of developing postoperative complication between surgery > 8 weeks after their COVID-19 infection and pre-COVID-19 group.

**Table 3:** The univariate analysis of postoperative complications

Characters	DVT		*P* value	Incision complication		*P* value	Overall complication		*P* value
	Yes	No		Yes	No		Yes	No	
Age			0.541			0.985			0.944
<50	12	437		11	438		24	425	
≥ 50	13	444		12	445		24	433	
Clinical N stage			0.094			0.116			0.600
cN0	12	559		10	561		26	545	
cN1	6	213		13	206		16	203	
cN2	5	63		0	68		3	65	
cN3	2	46		0	48		3	45	
Clinical T stage			0.627			0.661			0.352
cT1	14	476		12	478		29	461	
cT2	8	354		8	354		16	346	
cT3	3	26		3	26		3	26	
cT4	0	25		0	25		0	25	
Breast surgery			0.558			0.404			0.676
Mastectomy	18	624		17	625		35	607	
BCS	7	257		6	258		13	251	
Groups			0.024			0.088			0.033
Pre-COVID	4	325		6	323		10	319	
0-2w	6	45		3	48		9	42	
3-8w	12	324		12	324		24	312	
>8w	3	187		2	188		5	185	

**Table 4:** The multivariate logistic regression analysis of postoperative complications

Groups	DVT	Incision complication	Overall complication
	OR (95%CI)	OR (95%CI)	OR (95%CI)
Pre-COVID	Ref	Ref	Ref
0-2w	10.556 (1.095-303.313, *P* = 0.008)	3.365 (0.382–29.637, *P* = 0.009)	6.836 (1.691–27.632, *P* = 0.007)
3-8w	3.551 (1.020-14.674, *P* = 0.048)	2.055 (1.196–3.529, *P* = 0.274)	2.786 (1.136–5.808, *P* = 0.012)
>8w	1.216 (0.289–5.887, *P* = 0.125)	0.658 (0.324–1.336, *P* = 0.247)	1.069 (0.770–1.483, *P* = 0.691)

DVT: deep vein thrombosis; OR: odds ratio; CI: confidence interval

We analyzed the correlation between the severity of COVID-19 infection and postoperative complications. Although the results showed that there was a trend, the difference was not statistically significant (*P* = 0.497).

### The incidence of incision complication after surgery

Although patients with COVID-19 infection had a higher rate of incision complication when compared with pre-COVID-19 infection (2.95% vs. 1.82%), there was no significant difference (*P* = 0.088). Patients underwent surgery within 2 weeks after COVID-19 infection had the highest rates of incision complication (5.88%) when compared with other groups, while the incidence was decreased into 2.66% when surgery 2 weeks or more after their COVID-19 diagnosis date.

### The incidence of DVT after surgery

The incidence of postoperative DVT for each group are also detailed in Table 2. All DVT types were intermuscular venous thrombosis. Patients with peri-operative SARS-CoV-2 infection had the highest rates of DVT (11.76%) when compared with other groups, while the incidence was decreased into 2.85% when surgery 2 weeks or more after their COVID-19 diagnosis date. Patients with surgery 8 weeks or more after their COVID-19 diagnosis date had a similar incidence of postoperative DVT compared with the pre-COVID-19 group.

Patients infected with COVID-19 had a higher incidence of DVT after surgery compared to patients without COVID-19 infection (3.64% vs. 1.21%). The incidence of DVT was 3.57% in patients with surgery 3–8 weeks after their COVID-19 diagnosis date, and it was 4.65% in patients with surgery within 8 weeks after their COVID-19 diagnosis date.

The results of univariate analysis showed that the time period of surgery was associated with the incidence of DVT significantly. The incidence of DVT in patients with peri-COVID-19 infection was significantly higher than other groups. The multivariable logistic regression analysis demonstrated that timing of surgery was associated with DVT (OR = 2.795; 95% CI: 0.692–11.278, *P =* 0.024). Patients performed surgery within 2 weeks after COVID-19 infection had the highest risk of DVT (OR = 10.556; 95% CI: 1.095-303.313, *P =* 0.030) when compared to other groups.

### The result of follow-up

We made follow-up in COVID-19 infection patients with complication after surgery. The median follow-up was 10 months (9 to 12 months). The 21 patients with DVT were treated with an elevation of the affected limb, thrombolytic therapy and conventional anticoagulation. The 17 patients with incision complication were treated hemostasis by compression. All patients were recovered after continuous treatment. All patients completed the therapy. There were no serious complications and death, and no adverse effects on subsequent anti-tumor therapy.

It is suggested that the overall benefit of surgery after COVID-19 is greater than the risk of complications caused by COVID-19 infection, and the incidence of postoperative complication may be lower if preventive treatment is carried out.

## Discussion

In this paper, we assessed the association between the timing of surgery after COVID-19 infection and risk of postoperative complication in 577 patients who underwent major breast cancer operations. The incidence of overall complications was decreased into tolerance interval when surgery 2 weeks or more after their COVID-19 diagnosis date. At the same time, all patients with complications after surgery were safely recovered from treatment. It is suggested that the overall benefit of surgery after COVID-19 is greater than the risk of complication caused by COVID-19. Therefore, in the era of “Post COVID-19 Pandemic”, it needs to be cautious when elective breast cancer surgery was performed within 2 weeks after the COVID-19 infection (“peri-COVID-19”). Although the incidence of complications in patients undergoing surgery 2 weeks after COVID-19 infection is still slightly high, surgical treatment can be recommended considering the urgency of breast cancer treatment, the good prognosis of complications and the lack of influence on subsequent adjuvant therapy.

The incidence of post-surgery complications in patients infected with COVID-19 has been the focus of attention in many studies [8–10]. The study from United States showed that major elective surgery 0–4 weeks after COVID-19 infection is associated with an increased risk of postoperative complications, whereas complications were decreased when performed > 8 weeks after COVID-19 diagnosis [10]. However, a single-center study from India came to a different conclusion [14]. In the multivariate analysis, moderate and mild COVID-19 infection and surgery performed within 7 weeks of the onset of COVID-19 were not associated with postoperative morbidity. This study was conducted between May 2020 and November 2021, which is later than the previous study, and the virus strains may be different from other studies. As one of the postoperative complications, postoperative DVT is a venous disorder disease caused by abnormal blood clotting in deep veins, and the main adverse consequences are pulmonary embolism and post thrombotic syndrome, which can significantly affect the quality of life of patients and even lead to death [15]. The risk of DVT in breast cancer patients is higher than that in non-cancer patients. At present, the acceptable incidence of DVT after breast cancer surgery has not received an international consensus, and several studies have shown that the incidence of DVT after breast cancer surgery is 0.16-3.2% [16–19]. The study from COVID-19 Surg Collaboration showed that the incidence of postoperative DVT is 0.5% in non-COVID-19 patients, and 2.2% in patients within four weeks after COVID-19 infection [20].

To allow the use of ventilators, hospital space and personnel for COVID-19, patients elective surgery was cancelled and reduced, and cancer care was deprioritized and delayed. Over time this would clearly have led to a collateral increase in the number of deaths. Sgarzani R et al. [21] made the suggestions on risk stratification for breast cancer were developed: based on tumor stage and biology, patients were divided into those for whom surgery was time critical and those for whom surgery could be reasonably deferred for a period, like up to 60 days for early-stage breast cancer. Similar to the study of Sgarzani R et al. [21], our data also showed that the impact of pandemic on diagnostic and therefore therapeutic delay in patients with breast cancer. Delayed and later-stage diagnoses as well as uncertainties about the future and the fear of delays in radiotherapy (mandatory adjuvant treatment after BCS), may be responsible for the increased number of indications of mastectomy. For the axillary treatment of breast cancer, we recommend intraoperative One step nucleic amplification (OSNA) test as the study of Anedda G et al. [22]. The axillary treatment of breast cancer using the OSNA method and the ACOSOG Z0011 protocol resulted in a significant reduction in the volume of reoperations required for the radicalisation of metastatic LNs, allowing more interventions to be performed, thus reducing the length of the waiting list and the impact of the pandemic on surgical activity.

According to epidemiological testing, the vast majority of patients in our study were currently infected with the Omicron strain. The above studies all included multiple surgery types. In our study, we only focused on breast cancer surgery in order to provide treatment advice for breast cancer patients, including mastectomy or breast-conserving surgery, axillary-sentinel lymph node biopsy, and axillary lymph node dissection. The results also showed an increased incidence of postoperative complications in COVID-19 infection patients compared with negative patients. At the same time, most of the patients in this study had mild infections, which could account for the relatively low incidence of postoperative complications. In our study, the incidence of DVT is 1.21% in non-COVID-19 patients, and patients within 2 weeks after COVID-19 infection had the highest rates of DVT (11.76%). The incidence was decreased into tolerance interval when surgery 2 weeks or more after their COVID-19 diagnosis date. Although our study suggests that the incidence of DVT decrease to an acceptable level when surgery 2 weeks or more after their COVID-19 diagnosis date, we recommend that patients need to be individually management according to their condition. Considering the risk of DVT and different tumor types, we recommend that a delay beyond 4 weeks can be applied to all tumor types taken into consideration.

This study had several limitations. First, the single-center observational study design may be associated with patient selection bias. The number of patients in peri-operation was too small to match by propensity score. Further multicenter studies with a greater number of patients are needed to confirm the present results. Second, the number of moderate/severe cases was small, so the conclusion can only be applied to mild cases. Third, this study only focused on breast cancer surgery in order to provide treatment advice for breast cancer patients, which limit the broad application of the findings.

## Conclusion

In the era of “Post COVID-19 Pandemic”, it needs to be cautious when breast cancer surgery was performed within 2 weeks after mild COVID-19 infection. Although the incidence of complications in patients undergoing surgery 2 weeks after COVID-19 infection is still slightly high, surgery might be recommended considering urgency of treatment, good prognosis of complications and the lack of influence on subsequent adjuvant therapy.

## Data Availability

The datasets generated during and/or analyzed during the current study are available from the corresponding author on reasonable request.
